# Association of Magnesium Intake with High Blood Pressure in Korean Adults: Korea National Health and Nutrition Examination Survey 2007–2009

**DOI:** 10.1371/journal.pone.0130405

**Published:** 2015-06-15

**Authors:** Mi-Kyeong Choi, Yun Jung Bae

**Affiliations:** 1 Division of Food Science, Kongju National University, Yesan, Republic of Korea; 2 Division of Food Science and Culinary Arts, Shinhan University, Uijeongbu, Republic of Korea; The University of Tokyo, JAPAN

## Abstract

**Background:**

Magnesium is known to lower the risk of cardiovascular disease. However, studies on its relationship with hypertension, a single and common cause of various chronic diseases, are limited and their findings are not consistent. The purpose of the present study is to identify the relationship between magnesium intake and high blood pressure (HBP) risk in Koreans.

**Methods:**

This research is a cross-sectional study based on the 2007~2009 Korean National Health and Nutritional Examination Survey data. This study investigated 11,685 adults aged over 20 to examine their general characteristics, anthropometry and blood pressure. Daily magnesium intake was analyzed using the 24-hour dietary recall method. To calculate the odds ratio (OR) of HBP risk (130/85 mmHg or over) according to the quartile of magnesium intake (mg/1000kcal) together with its 95% confidence interval (CI), multivariable logistic regression analysis was performed.

**Results:**

No significant association between dietary magnesium intake and the risk of HBP was found. In obese women, particularly, after adjusting relevant factors, the adjusted odds ratio of HBP prevalence in the highest magnesium intake quartile was 0.40 compared with the lowest magnesium intake quartile (95% CI = 0.25~0.63, P for trend = 0.0014). Women, especially obese women, were found to have a negative relationship of magnesium intake with HBP.

**Conclusions:**

The present results indicate that sufficient magnesium intake could be useful in decreasing the high blood pressure risk of obese women.

## Introduction

Cardiovascular diseases (CVD) are caused by abnormalities in the heart and blood vessels and mainly include conditions such as coronary heart disease, stroke, hypertension, peripheral vascular disease, rheumatic heart disease and heart failure [1]. Hypertension is a significant public health concern with world-wide distribution and is also the most common cardiovascular disease risk factor [2]. In Korea, it is also reported that CVD accounts for 9.5% of total causes of death [3], and the prevalence rate of hypertension (systolic blood pressure (SBP) above 140 mmHg or diastolic blood pressure (DBP) of 90 mmHg / taking antihypertensive medication) of adults above 30 is 31.5% [4], thus, the risk of high blood pressure is quite severe. However, it is believed that hypertension rarely presented clear symptoms until blood pressure goes extremely high with concomitant complications.

The number of hypertension patients is steadily growing, and controlling high blood pressure and identifying its prevention factors are urgent tasks. Various dietary factors, such as obesity, aging, decreased physical activity and excessive sodium intake, are one of the causes of high blood pressure. Based on strong evidence, excessive sodium intake is a causal risk factor for hypertension, whereas a diet rich in fruit, vegetables, and low-fat dairy products and low in sodium and saturated fat has been recommended to prevent and reduce hypertension [5]. It is reported that the consumption of plant foods, in particular, leads to a reduction in systolic blood pressure [6]. Plant foods may confer beneficial effects on blood pressure control through their rich array of nutrients and constituents (e.g., fiber, magnesium, potassium, and other food components) and on satiety, body mass, and insulin sensitivity [6].

Magnesium is a major mineral that exists in the human body at a level of approximately 25 g. Magnesium, as a constituent of chlorophyll, is contained in large quantities of green leafy vegetables. In particular, magnesium given in conjunction with taurine lowers blood pressure, improves insulin resistance, retards atherogenesis, prevents arrhythmias, and stabilizes platelets [7]. In addition, a previous study reported that with regard to CVD diseases, magnesium prevents calcification of atherosclerotic plaques, and self-reported magnesium intake has an inverse correlation with arterial calcification [8]. Various epidemiologic, observational, and clinical trial data show that a diet high in magnesium (at least 500~1000 mg/day) lowers blood pressure; however, the conclusions are not consistent, and these studies are mostly conducted in Western countries, where plant food intake is not so popular [9,10].

Koreans have kept their traditional diet pattern, which consists mostly of plant foods, and the intake rate of plant food is up to 79.4% [4]. Therefore, considering that magnesium is mainly supplied through plant food, magnesium deficiency has not been a concern. However, according to the recent analysis of magnesium intake amount of Korean adults, men showed intakes of 301~321 mg/day (the rates of subjects with magnesium intakes lower than the EAR: 50.0~57.4%) and women 230~247 mg/day (the rates of subjects with magnesium intakes lower than the EAR: 53.5~62.6%) [11].

Although it is reported that magnesium, which is supplied primarily through plant food, is related to high blood pressure due to its metabolic function, there are not many comprehensive studies of magnesium’s relation to high blood pressure in Koreans, who consume plant food in high quantities. Therefore, the present study was conducted to investigate the association between dietary magnesium intake and the high blood pressure risk. We hypothesized that lower dietary magnesium intake would correlate with a risk of high blood pressure in the general population.

## Subjects and Methods

### Study population

The data analyzed in this study were obtained from the Korean National Health and Nutritional Examination Survey (KNHANES) performed between 2007 and 2009, which used a rolling sampling design involving a complex, stratified, multistage, probability-cluster survey of a representative sample of the non-institutionalized civilian Korean population [12]. The survey data were compiled through a health interview (physical examination), health examination (anthropometric measurements and blood pressure measurement), and a nutrition survey. The KNHANES received ethical approval by the Institutional Review Board of the Korea Centers for Disease Control and Prevention (KCDC) (IRB No: 2007-02-CON-04-P, 2008-04EXP-01-C, 2009-01CON-03-2C), and written consent was obtained from all of the participants. In addition, this survey was conducted according to the guidelines laid down in the Declaration of Helsinki.

Of 15,077 NHANES participants during 2007~2009 who were ≥20 years of age and gave reliable information on the nutrition survey, we excluded participants who did not report their diagnosed hypertension status or who reported taking antihypertensive medication (n = 3,019), those who were pregnant (n = 102), those who did not provide socio-demographics information (smoking, alcohol intake, and physical activity) (n = 87), those with missing blood pressure data (n = 16), and those who reported implausible dietary intakes (lower than 500 kcal/day or greater than 5,000 kcal/day) (n = 168). Finally, 11,685 subjects aged 20 years and older were included in the analysis.

### General Characteristics, Anthropometrics, and Blood Pressure Measurements

The health examination included anthropometric measurements (height, body weight, and body mass index (BMI)) and blood pressure measurements. In the health interview, a questionnaire including information on sex, age, smoking history, alcohol intake, physical activity, education level, income, and menopausal status was administered by trained interviewers. Metabolic Equivalent of Task values (METs) were used to classify physical activity as low, middle, or high. METs are multiples of the resting metabolic rates and were calculated using the short form (version 2.0, April 2004) of the International Physical Activity Questionnaire.

Height was measured within 0.1 cm, and body weight was measured with a metric weight scale to the nearest 0.1 kg in light clothing without shoes. BMI was calculated as body weight in kilograms divided by height in meters squared. Three blood pressure measurements were taken at the mobile examination center by a trained and certified observer following the KNHANES protocol. Systolic and diastolic blood pressures (SBP and DBP) were three times on the right arm while the individual was in a seated position after at least 5 min of rest using a standard sphygmomanometer. The average of the second and third measurements for both SBP and DBP was used in the analysis.

### Dietary Assessment and Magnesium Database

Well trained dietary interviewers from the Korea Health Industry Development Institute surveyed each subject to collect dietary information through 24-hour dietary recalls. The daily magnesium intakes from foods were estimated using a magnesium content database produced by our previous studies [13] and the food composition table of the National Rural Living Science Institute, Korea [14]. The subjects of the 2007~2009 KNHANES consumed 2,429 foods based on 24-h dietary recall data. Regarding food intake, 59.3% of the items had information on magnesium content in the database. Foods not included in the magnesium content database were calculated by replacing them with similar foods in the database.

Dietary nutrient intakes were assessed by the provided energy and 11 nutrients based on the dietary reference intakes for Koreans. From these data, we selected nutrients including energy intake, carbohydrate, protein and fat intake as a percentage of energy, and crude fiber, calcium, sodium and potassium intake.

### Statistical Analysis

All statistical analyses were performed using SAS software (version 9.3, SAS Institute Inc., Cary, NC, USA) through a “survey procedure”. The participants’ characteristics were compared according to the magnesium intake (mg/1000 kcal) quartiles. Data were presented as mean±SE or as percentage (% and SE) and were age-adjusted. Multivariable logistic regression analysis was performed to estimate the odds ratio (OR) and 95% confidence interval (CI) of high blood pressure according to the magnesium intake quartiles, using the lowest quartile as the reference. High blood pressure was defined as SBP readings over 130 mmHg, or DBP over 85 mmHg. Although the typical diagnostic criterion for hypertension is SBP over 140 mmHg or DBP over 90 mmHg, the criterion of high blood pressure for diagnosing metabolic syndrome was used so that the correlation with magnesium intake at an elevated blood pressure level before disease morbidity could be analyzed [15].

In addition, when analyzing the risk of high blood pressure by magnesium intake level through regression analysis in this study, an intake amount of magnesium per 1000 kcal was analyzed to exclude the risk of the conclusion being affected by the total energy intake. Indeed, when classifying the group according to raw intake of magnesium, a significant difference of energy intake by groups existed. ORs were initially calculated following adjustment for age (continuous), sex, and BMI (continuous) in model 1. In model 2, variables that were further adjusted for included education (elementary school or less, middle or high school, college or more), monthly household income (quartiles of household income), and physical activity (MET). Smoking status (never smoker, past smoker, current smoker) and frequency of alcohol consumption (none, <1 time/month, 2~4 times/month, ≥2 times/week) were also adjusted. In addition, dietary factors such as total energy (kcal/day), carbohydrate (%energy), total fat (%energy), fiber (g/1000 kcal), calcium (mg/1000 kcal), and sodium (mg/1000 kcal) intakes were adjusted for as continuous variables in model 3.

Moreover, because the OR of having high blood pressure according to magnesium intake may be different between men and women, an analysis based on sex was performed. Because obesity is related to high blood pressure [16], the OR of high blood pressure according to obesity level by sex (BMI 25 kg/m^2^, as standard) was also calculated. The reported probability values were 2-sided and a p < 0.05 was considered to be statistically significant.

## Results

The general characteristic and daily dietary intakes by quartile of dietary magnesium intake are shown in Table 1. The mean magnesium intake was <107.73 mg/1,000 kcal in the lowest quartile and ≥177.70 mg/1,000 kcal in the highest quartile. No difference was observed in BMI among the quartiles, whereas age in the higher quartiles was significantly higher than that of the lowest quartiles (p<0.0001). Participants with higher magnesium intake had a significantly lower current smoking rate (p<0.0001) and drinking rate (p = 0.0006), whereas education level and household income in the highest quartile were significantly higher than those in the lowest quartile (p<0.0001). The total energy intake was significantly lower in the higher quartiles of magnesium intake (Table 1). The intakes of carbohydrate (%energy), protein (%energy), fiber (g/1000 kcal), calcium (mg/1000 kcal), sodium (mg/1000 kcal), and potassium (mg/1000 kcal) were higher in the higher quartiles (p<0.0001). Total fat intake (%energy) was not significantly different among the quartiles.

**Table 1:** Characteristics of participants according to magnesium intake.

Variables	Magnesium intake				P value
	Quartile 1	Quartile 2	Quartile 3	Quartile 4	
Magnesium intake (range, mg/1000kcal)	<107.7	107.7–138.8	138.8–177.7	≥177.7	
No. of subjects	2885	2906	2888	3006	
Male (%)	54.4	50.8	51.2	44.7	<.0001
Age^a^(years)	40.4±0.4	41.5±0.4	42.3±0.3	44.2±0.3	<.0001
BMI (kg/m^2^)	23.5±0.1	23.3±0.1	23.4±0.1	23.5±0.1	0.1560
Education (%)					
≤Elementary	16.9±0.9	14.8±0.8	13.2±0.7	15.3±0.8	<.0001
Middle/High	55.2±1.2	51.2±1.2	52.1±1.2	52.6±1.2	
≥College	27.9±1.1	34.0±1.2	34.7±1.3	32.1±1.3	
Household income (%)					
Quartile 1 (Low)	17.7±1.1	12.3±0.8	10.7±0.7	12.3±0.8	<.0001
Quartile 2	27.4±1.3	25.5±1.1	23.5±1.1	23.3±1.2	
Quartile 3	28.5±1.2	30.5±1.3	32.3±1.1	29.8±1.2	
Quartile 4 (High)	26.4±1.3	31.8±1.3	33.5±1.3	34.6±1.6	
Physical activity^b^ (%)					
Low	50.6±1.2	47.9±1.2	50.1±1.2	45.7±1.2	0.0429
Moderate	45.8±1.2	48.3±1.2	45.6±1.2	50.3±1.1	
High	3.6±0.5	3.8±0.4	4.4±0.5	4.1±0.5	
Smoker (%)					
Never	46.9±1.1	52.6±1.2	53.3±1.1	57.5±1.1	<.0001
Past	19.6±1.0	18.3±0.8	20.3±0.9	19.1±0.9	
Current	33.5±1.1	29.1±1.2	26.3±1.0	23.4±1.0	
Frequency of alcohol (%)					
None	20.9±0.8	19.4±0.9	22.5±1.0	23.9±1.0	0.0006
<1 time/month	27.4±1.1	29.7±1.0	30.0±1.0	30.6±1.0	
2~4 times/month	25.6±1.1	27.1±1.0	25.4±1.1	24.1±1.0	
≥2 times/week	26.1±1.1	23.8±1.0	22.1±0.9	21.4±1.0	
Daily dietary intakes					
Total energy (kcal)	2022.2±20.5	1971.4±18.4	1889.5±18.1	1742.6±15.8	<.0001
Carbohydrate (%Energy)	65.7±0.4	67.4±0.3	67.8±0.3	67.9±0.3	<.0001
Fat (%Energy)	16.4±0.2	16.4±0.2	16.7±0.2	16.7±0.2	0.4612
Protein (%Energy)	11.9±0.1	13.6±0.1	14.6±0.1	16.6±0.1	<.0001
Fiber (g/1000kcal)	2.9±0.0	3.7±0.0	4.3±0.1	5.4±0.1	<.0001
Calcium (mg/1000kcal)	179.6±2.5	230.6±2.8	280.0±3.1	362.5±3.6	<.0001
Sodium (mg/1000kcal)	1989.0±21.2	2425.3±24.4	2758.3±23.9	3417.4±38.3	<.0001
Potassium (mg/1000kcal)	1176.9±10.0	1470.5±8.6	1698.5±9.7	2061.7±15.7	<.0001

Data represent age-adjusted mean or prevalence (%)±SE, except for age.

a Non-adjusted values.

b Defined as low (<600 MET-minutes per week), moderate (≥600 to <3000 MET-minutes per week), and high (≤3000 MET-minutes per week) levels of physical activity.

The relationships between magnesium intake and the risk of high blood pressure by gender are shown in Table 2. Among all of the participants, dietary magnesium was inversely associated with the risk of high blood pressure (Model 1: 4th vs. 1st quartile, OR = 0.75, 95% CI = 0.64~0.87, P for trend = 0.0009), but there was no significant relation between dietary magnesium intake and high blood pressure risk after adjusting for potential confounders (Model 3). The intake of magnesium did not have a significant difference on OR for the risk of high blood pressure in both men and women. Considering the close correlation of obesity and high blood pressure, the OR of high blood pressure according to magnesium intake by obesity level was analyzed (Table 3). In the case of normal weight, magnesium intake amount did not have a significant difference on OR for the prevalence of high blood pressure in both men and women. However, in the case of obese women, subjects who ingested more magnesium had a lower OR for the risk of high blood pressure after adjusting for various factors (Model 3: 4th vs. 1st quartile, aOR = 0.40, 95% CI = 0.25~0.63, P for trend = 0.0014).

**Table 2:** Multivariate odds ratio for high blood pressure according to magnesium intake.

Variables	Magnesium intake				*p* for trend
	Quartile 1	Quartile 2	Quartile 3	Quartile 4	
Total					
No. of HBP subjects (%)	768 (23.6)	718 (23.2)	669 (22.2)	634 (21.4)	
Male/Female (n)	404/364	407/311	387/282	323/311	
Mg Intake range (mg/1000kcal)	<108.2	108.2–139.5	139.5–178.6	≥178.6	
Model 1^a^	1.00	0.98 (0.84–1.14)	0.89 (0.76–1.04)	0.75 (0.64–0.87)	0.0009
Model 2^b^	1.00	1.00 (0.86–1.17)	0.96 (0.82–1.13)	0.81 (0.69–0.95)	0.0323
Model 3^c^	1.00	1.03 (0.88–1.20)	1.00 (0.84–1.19)	0.87 (0.71–1.05)	0.3176
Male					
No. of HBP subjects (%)	356 (27.3)	409 (32.0)	377 (29.7)	379 (30.3)	
Mg Intake range (mg/1000kcal)	<104.2	104.2–136.1	136.1–170.9	≥170.9	
Model 1^d^	1.00	1.25 (1.02–1.54)	1.09 (0.88–1.35)	0.99 (0.79–1.23)	0.0693
Model 2^e^	1.00	1.27 (1.02–1.57)	1.15 (0.92–1.45)	1.03 (0.83–1.29)	0.1065
Model 3^f^	1.00	1.31 (1.05–1.63)	1.21 (0.94–1.56)	1.10 (0.82–1.46)	0.0853
Female					
No. of HBP subjects (%)	395 (17.8)	306 (14.3)	279 (14.0)	288 (14.7)	
Mg Intake range (mg/1000kcal)	<111.0	111.0–142.4	142.4–183.7	≥183.7	
Model 1^d^	1.00	0.80 (0.64–1.02)	0.73 (0.58–0.92)	0.71 (0.57–0.89)	0.0137
Model 2^e^	1.00	0.80 (0.63–1.02)	0.74 (0.59–0.93)	0.72 (0.57–0.91)	0.0224
Model 3^f^	1.00	0.80 (0.63–1.02)	0.74 (0.58–0.95)	0.72 (0.54–0.97)	0.0912

HBP: High Blood Pressure

a Model 1: Adjusted for age, sex, BMI (continuous).

b Model 2: Adjusted for education (elementary school or less, middle or high school, college or more), income (quartiles of household income), physical activity (MET), smoking status (never smoker, past smoker, current smoker), and frequency of alcohol (none, <1 time/month, 2~4 times/month, ≥2 times/week) in addition to model 1.

c Model 3: Adjusted for total energy (kcal/day), carbohydrate (%energy), proteins (%energy), fat (%energy), fiber (g/1000 kcal), calcium (mg/1000 kcal), sodium (mg/1000 kcal), and potassium (mg/1000 kcal) intakes as continuous variables in addition to model 2.

d Model 1: Adjusted for age and BMI (continuous).

e Model 2: Adjusted for education (elementary school or less, middle or high school, college or more), income (quartiles of household income), physical activity (MET), smoking status (never smoker, past smoker, current smoker), and frequency of alcohol (none, <1 time/month, 2~4 times/month, ≥2 times/week) in addition to model 1.

f Model 3: Adjusted for total energy (kcal/day), carbohydrate (%energy), proteins (%energy), fat (%energy), fiber (g/1000 kcal), calcium (mg/1000 kcal), sodium (mg/1000 kcal), and potassium (mg/1000 kcal) intakes as continuous variables in addition to model 2.

**Table 3:** Multivariate odds ratio for high blood pressure according to magnesium intake by obesity and menopause.

Variables	Magnesium intake				*p* for trend
	Quartile 1	Quartile 2	Quartile 3	Quartile 4	
Male					
Obesity (≥25kg/m^2^)					
No. of HBP subjects (%)	131 (36.4)	184 (45.0)	176 (41.6)	148 (40.0)	-
Mg Intake range (mg/1000kcal)	<102.6	102.6–137.0	137.0–174.0	≥174.0	-
Model 1^a^	1.00	1.43 (1.01–2.01)	1.22 (0.85–1.73)	1.05 (0.72–1.52)	0.1344
Model 2^b^	1.00	1.36 (0.95–1.93)	1.29 (0.89–1.88)	1.06 (0.72–1.56)	0.2361
Model 3^c^	1.00	1.42 (0.98–2.06)	1.40 (0.92–2.14)	1.20 (0.72–1.99)	0.2101
Normal weight (<25kg/m^2^)					
No. of HBP subjects (%)	228 (23.0)	222 (25.3)	211 (24.0)	221 (24.3)	-
Mg Intake range (mg/1000kcal)	<105.4	105.4–135.5	135.6–170.6	≥170.6	-
Model 1^a^	1.00	1.09 (0.84–1.41)	1.01 (0.77–1.32)	0.91 (0.68–1.21)	0.6099
Model 2^b^	1.00	1.14 (0.87–1.50)	1.06 (0.79–1.41)	0.96 (0.71–1.29)	0.6037
Model 3^c^	1.00	1.14 (0.86–1.51)	1.06 (0.78–1.45)	0.93 (0.64–1.35)	0.5467
Female					
Obesity (≥25kg/m^2^)					
No. of HBP subjects (%)	150 (31.2)	127 (26.4)	108 (24.5)	82 (17.8)	-
Mg Intake range (mg/1000kcal)	<112.2	112.2–146.4	146.4–191.8	≥191.8	-
Model 1^a^	1.00	0.79 (0.56–1.13)	0.69 (0.47–1.00)	0.45 (0.32–0.64)	<.0001
Model 2^b^	1.00	0.81 (0.56–1.16)	0.70 (0.48–1.03)	0.46 (0.33–0.66)	0.0002
Model 3^c^	1.00	0.76 (0.53–1.10)	0.64 (0.41–0.99)	0.40 (0.25–0.63)	0.0014
Normal weight (<25kg/m^2^)					
No. of HBP subjects (%)	247 (13.7)	187 (11.3)	163 (10.4)	199 (13.8)	-
Mg Intake range (mg/1000kcal)	<110.6	110.6–141.6	141.6–181.9	≥181.9	-
Model 1^a^	1.00	0.87 (0.65–1.16)	0.83 (0.55–0.98)	0.94 (0.70–1.25)	0.1607
Model 2^b^	1.00	0.85 (0.63–1.14)	0.72 (0.54–0.98)	0.93 (0.69–1.25)	0.1533
Model 3^c^	1.00	0.88 (0.66–1.19)	0.77 (0.56–1.06)	1.02 (0.71–1.47)	0.1980
Menopause					
Premenopause					
No. of HBP subjects (%)	102 (9.5)	92 (8.0)	94 (7.9)	105 (8.9)	-
Mg Intake range (mg/1000kcal)	<110.6	110.6–140.6	140.6–181.1	≥181.2	-
Model 1^a^	1.00	0.73 (0.51–1.03)	0.68 (0.49–0.95)	0.69 (0.49–0.97)	0.0867
Model 2^b^	1.00	0.70 (0.49–1.01)	0.70 (0.50–0.97)	0.69 (0.49–0.98)	0.1082
Model 3^c^	1.00	0.70 (0.48–1.00)	0.70 (0.48–1.00)	0.67 (0.43–1.04)	0.1489
Postmenopause					
No. of HBP subjects (%)	304 (37.5)	232 (32.0)	195 (29.9)	144 (25.9)	-
Mg Intake range (mg/1000kcal)	<113.2	113.2–148.7	148.7–195.1	≥195.1	-
Model 1^a^	1.00	0.87 (0.65–1.16)	0.80 (0.60–1.08)	0.66 (0.49–0.88)	0.0367
Model 2^b^	1.00	0.90 (0.67–1.20)	0.83 (0.61–1.11)	0.66 (0.49–0.88)	0.0405
Model 3^c^	1.00	0.92 (0.68–1.24)	0.85 (0.60–1.20)	0.70 (0.47–1.05)	0.3811

HBP: High Blood Pressure

a Model 1: Adjusted for age and BMI (continuous).

b Model 2: Adjusted for education (elementary school or less, middle or high school, college or more), income (quartiles of household income), physical activity (MET), smoking status (never smoker, past smoker, current smoker), and frequency of alcohol (none, <1 time/month, 2~4 times/month, ≥2 times/week) in addition to model 1.

c Model 3: Adjusted for total energy (kcal/day), carbohydrate (%energy), protein (%pro), fat (%energy), fiber (g/1000 kcal), calcium (mg/1000 kcal), sodium (mg/1000 kcal), and potassium (mg/1000 kcal) intakes as continuous variables in addition to model 2.

## Discussion

Based on the KNHANES analysis, the relationship between magnesium intake and high blood pressure risk was investigated in this study. There was no significant relation between dietary magnesium intake and high blood pressure risk after adjusting for potential confounders. However, when the obesity group and the normal-weight group were separated in the analysis, the obese female group, in particular, showed a significant negative correlation between magnesium intake and high blood pressure risk.

Many studies report that a high magnesium intake ranging from 500 to 1000 mg/day may reduce blood pressure [9,10,17]. It is known that magnesium lowers blood pressure by functioning as a calcium channel blocker. In other words, magnesium competes with the sodium binding site of smooth muscle cells, increases prostaglandin E, interacts with potassium, induces vasodilation, reduces intra-cell calcium and sodium contents and decreases blood pressure [18,19]. The negative relationship between magnesium intake and blood pressure is reported in epidemiological studies and observational studies. Song et al. [20] examined 28,349 middle-aged women in a follow-up observational study over a 10-year period and reported that the highest quartile of magnesium intake (434 mg/day) showed significantly lower hypertension risk than the lowest quartile (356 mg/day), which indicated that magnesium intake had a significant negative correlation with hypertension prevalence. In this study, women’s magnesium intake was inversely related to the risk of high blood pressure. However, after adjusting for several confounding factors, the result was attenuated. Especially when potassium intake, one of the confounding factors, was adjusted, the inverse relationship of magnesium intake and the risk of high blood pressure did not appear. Green leafy vegetables richly contain magnesium and trace amounts of potassium. This seems to affect potassium intake, which in turn affects the relationship between magnesium intake and the risk of high blood pressure.

Ascherio et al. [21] reported that in women with hypertension, magnesium intake showed no significant correlation with hypertension risk. However, in women with normal-range blood pressure, the authors found a significant negative correlation between magnesium intake and blood pressure after adjusting for age, BMI, alcohol and energy intakes. The present study, based on the standard criteria for a hypertension diagnosis of 140/90 mmHg or over, found no significant relationship between magnesium intake and hypertension prevalence (data not shown). However, based on the criteria of 130/85 mmHg or over, which is used for metabolic syndrome diagnosis [15], magnesium intake showed a significant negative relationship with high blood pressure risk. The study findings are meaningful in signaling that, just as in previous studies, magnesium intake through daily meals plays an important role in high blood pressure prevention.

It has been reported that supplementing magnesium has inconsistent effects in lowering blood pressure compared with magnesium intake through daily meals. Itoh et al. [22] found a blood pressure decrease in 33 healthy Japanese who supplemented with magnesium (male 548 mg/day, female 411 mg/day). In contrast, Cappucio et al. [23] reported that they found no significant change in blood pressure when supplementing daily magnesium of 15 mmol for 17 patients with mild hypertension. Such inconsistent results of magnesium supplementation on hypertension can be explained due to the interaction among research participants’ groups, the intervention period, and interaction with other nutrients or medications. These inconsistent research findings preclude the use of magnesium supplement treatment in clinical trials [24].

Nevertheless, Houston and Harper [25] emphasized magnesium intake for hypertension prevention or health improvement in the community. Klevay and Milne [26] reported that the current recommended magnesium level of 320 mg/day is appropriate for healthy cardiovascular functioning. In Western countries, magnesium intake has decreased continuously from 500 mg/day in the 1900s to the current 175 mg/day [27]. Accordingly, the number of people with magnesium deficiency has also increased. Magnesium is a component of chlorophyll and is contained in many green leafy vegetables. Consequently, magnesium intake seems to be sufficient in the Korean diet style, which is centered on plant foods [13,28]. In Korea, the recommended magnesium intake for men aged between ages 19 and 20 is 340 mg; men aged 30 and over, 350 mg; and women aged 10 and over, 280 mg [29]. In the present study, the magnesium intake showing a negative relationship with high blood pressure risk in women was 183.7 mg/1000kcal/day in the highest quartile. In this study, daily total magnesium amount exceeds the recommended magnesium intake level. Other studies assessing daily magnesium intake found that 60 post-menopausal women had a daily intake of 345.9 mg [30], exceeding the recommended level whereas 30 women living in rural locations had 259.1 mg [31]; 139 adult women, 259.1 mg [13]; and 294 adult women, 85% of the recommended level, failing to meet the established reference criteria [32]. Therefore, to prevent or lower the risk of hypertension, it is deemed necessary to increase magnesium intake through daily meals. In the present study, magnesium intake was analyzed by considering energy intake (mg/1000 kcal). Accordingly, increasing magnesium density in the diet seems particularly effective in reducing high blood pressure risk.

In this study, the negative relationship between magnesium intake and high blood pressure risk was found in obese women only. To date, no research has been performed that explains such a mechanism, and each study has found only inconsistent outcomes. Nevertheless, Zhang et al. [33] reported in the JACC study examining 58,615 Japanese that an association of magnesium intake with mortality rate due to congestive heart failure appeared only in women. To investigate whether the present study’s findings were possibly due to hormonal functions, we separated pre-menopausal women from post-menopausal women to analyze the relationship between magnesium intake and high blood pressure risk. Consequently, in the post-menopausal group, after adjusting for age, BMI, educational level, physical activities, smoking and drinking status, the aOR for high blood pressure risk of the highest magnesium intake quartile was significantly lower than that of the lowest quartile. However, after adjusting for energy, carbohydrate, protein, fat, fiber, calcium, sodium and potassium intakes related to blood pressure, magnesium intake showed no significant relationship with high blood pressure risk. Such a result indicates that the negative relationship in women between magnesium intake and high blood pressure risk was not primarily due to female hormones. Further research to elucidate this issue is required.

In addition, the relationship between magnesium intake and the risk of high blood pressure differs between sexes. Similarly, sodium sensitivity, one of the main dietary factors that affect high blood pressure, also differs between men and women [34–36]. In particular, the absolute amount of sodium intake is lower in women than in men, and sodium sensitivity is higher in women than in men [35]. Therefore, as complementary measures, the relationship between magnesium intake and risk of high blood pressure was analyzed separately between men and women, and the amount of sodium intake was set as the confounding factor for analysis. However, we cannot precisely explain the difference in the relationship between magnesium intake and the risk of high blood pressure between men and women.

Epidemiological studies have suggested that sufficient magnesium intake could help lower the risk of metabolic syndrome [37]. He et al. [38] observed 4,637 US adults aged between 18 and 30 in a follow-up study and found that the metabolic syndrome risk of the highest magnesium intake quartile was significantly lower than that of the lowest quartile (aOR = 0.69, 95% CI = 0.52~0.91, P for trend<0.01). In addition, the authors showed that the individual metabolic syndrome diagonal indexes centering on insulin resistance had a significant negative relationship with magnesium intake. Liu et al. [39] examined 37,183 women in a randomized, double-blind, placebo-controlled trial and found that dairy product intake had a relationship with diabetes risk reduction. Their interpretation of the result was that because dairy products had high contents of magnesium, calcium, lactose and milk protein, they increased the feeling of fullness more than other food or beverages containing more sugar and therefore reduced the risk of overweight and obesity. Obesity is one of the diagonal indexes for metabolic syndrome and an independent and crucial risk factor of various diseases. Therefore, the present study analyzed the relationship between magnesium intake and high blood pressure risk according to the obesity levels of male and female participants. Consequently, in obese women, after adjusting for obesity-related factors, the high blood pressure risk of the highest magnesium intake quartile was significantly lower compared to that of the lowest quartile (aOR = 0.40, 95% CI = 0.25~0.63, P for trend = 0.0014). Such a result indicates that in obese women, sufficient magnesium intake could be effective in preventing or controlling high blood pressure.

The present study has some limitations in generalizing its findings. First, it did not perform the follow-up observation for high blood pressure risk according to magnesium intake levels and only analyzed these data using a cross-sectional design. Thus, the study findings may have a lower reliability than that of a prospective design despite the adjustment for interference factors. Second, as mentioned above, the hypertension diagnosis criterion in the present study was lowered from 140/90 mmHg or over to 130/85 mmHg or over for the purpose of protection. Therefore, any direct comparison with previous studies would be difficult. Third, following the present KNHANES in which the 24-hour dietary recall method was utilized to investigate the dietary intake levels, this study also used the method to analyze magnesium intake. Thus, it is possible that this research does not reflect the long-term magnesium intake status. However, because magnesium is a microelement, to reduce the risk of underestimating or overestimating its intake level in the food frequency questionnaire investigation, we used a magnesium content database among the diverse food data to accurately assess the intake levels. Ultimately, the present study has a strength in that it is the first study in Korea to identify the relationship between magnesium intake and high blood pressure risk in a large cohort of Koreans living in a mixed dietary environment, i.e., a traditional diet focused on plant foods with high magnesium content together with a Westernized diet.

In conclusion, this study found a negative relationship between magnesium intake and high blood pressure in obese women. These findings suggest that sufficient magnesium intake may reduce high blood pressure risk in obese women.
